# Prion Protein Codon Implicated in Resistance in Dogs Detected in Non-Domesticated Members of Mammalia

**DOI:** 10.3390/ani16071059

**Published:** 2026-03-31

**Authors:** Emily A. Wright, Vivienne A. Lacy, Georgina G. Brugette, Savannah M. Roberts, Emma K. Brookover, Daniel M. Hardy, Robert D. Bradley

**Affiliations:** 1 Independent Researcher, Rockwall, TX 75032, USA; 2 Department of Biological Sciences, Texas Tech University, 2901 Main Street, Lubbock, TX 79409, USA; vivi.lacy@gmail.com (V.A.L.); savmrob2001@gmail.com (S.M.R.); robert.bradley@ttu.edu (R.D.B.); 3 Natural Science Research Laboratory, Museum at Texas Tech University, 3301 4th Street, Lubbock, TX 79409, USA; brugettegeorgina@gmail.com; 4 Climate Center, Texas Tech University, Weeks Hall, 2508 Broadway Ave, Lubbock, TX 79409, USA; emma.brookover@tamuk.edu; 5 Department of Cell Biology and Biochemistry, Texas Tech University Health Sciences Center, Lubbock, TX 79430, USA; daniel.hardy@ttuhsc.edu

**Keywords:** canid, chiropteran, genetics, prion protein, susceptibility, transmissible spongiform encephalopathy

## Abstract

Transmissible spongiform encephalopathies (TSE) are fatal neurodegenerative diseases caused by the misfolding of proteins generated in the exon 3 region of the prion protein gene (*PRNP*). Recent investigations using protein misfolding cyclic amplification assays indicated that some canids displayed a low susceptibility to TSE due to a specific nonsynonymous substitution (human: N159D/E; canid: N163D/E; alignment herein: N302D/E) in the prion protein that may confer protection against prion seeding activity and propagation. To examine the molecular evolution underlying this observation, we determined the mammalian taxonomic distribution of the N159D/E substitution in 882 *PRNP* sequences representing 26 Orders, 132 families, and 686 species. Two families each in Carnivora (Canidae and Mustelidae) and Chiroptera (Mormoopidae and Vespertilionidae), and one family each in Artiodactyla (Moschidae) and Rodentia (Erethrizontidae), possessed N159D/E that has been reported to confer resistance to TSEs. Although no direct evidence linked a pattern of resistance (phylogenetic relatedness, geographic location, etc.) in these diverse species, it may be that coevolutionary pressures led 53 of the examined 686 species (1 domestic species, 52 wild species) to possess N159D/E. Therefore, the presence of N159D/E may not be the only factor in determining sensitivity to prion diseases; consequently, a more detailed investigation into the 53 species, such as knockout experiments, is warranted.

## Simple Summary

We examined the genetic variation in exon 3 of the prion protein gene across mammals as it pertains to potential resistance (i.e., limited susceptibility) to prion disease. Previous research suggests that a single codon reported in canids, N163D/E, confers protection against prion diseases in vitro. We used bioinformatics and phylogenetic analyses to discover that two families in Carnivora (Canidae and Mustelidae) and Chiroptera (Mormoopidae and Vespertilionidae) and one family in Artiodactyla (Moschidae) and Rodentia (Erethrizontidae) possessed the N163D/E substitution. The comprehensive molecular survey herein for mammalian candidates that may display a lack of sensitivity to prion diseases presents an update to the ever-growing field of prion disease. In vitro studies are needed to include these other species to truly reveal their susceptibility to prion disease and the role of N163D/E in mammalian species.

## 1. Introduction

Spongiform encephalopathies (SE) are fatal neurodegenerative diseases caused by a misfolding in the prion protein region encoded by exon 3 of the gene (*PRNP*). In humans, known prion diseases, including transmissible spongiform encephalopathies (TSE), are Creutzfeldt-Jakob disease (CJD), variant Creutzfeldt-Jakob disease, Kuru or Gertsmann-Sträussler-Scheinker disease (GSS), fatal and sporadic familial insomnia and variable protease-sensitive prionopathy (VPSPr) [1]. Several prion diseases have been identified in mammalian species, including scrapie in sheep and goats [2], chronic wasting disease (CWD) in cervids (deer species), bovine spongiform encephalopathy (BSE) in cattle, transmissible mink encephalopathy (TME), feline spongiform encephalopathy (FSE), and camel prion disease (CPD) [3,4,5,6,7,8].

The prion protein amino acid sequence (PrP) is highly conserved across mammalian species, with approximately 85% sequence identity to humans [9]. Substitutions in this highly conserved region may impact molecular folding processes and typically result in disease [10]. Normal cellular PrP (PrP^C^) is alpha-helical, whereas the infectious conformation (PrP^Sc^) is richer in beta-sheets formed post-translationally. Further, the infectious PrP^Sc^ tend to aggregate, serve as a template for generating new PrP^Sc^, and become resistant to proteases, leading to TSE [11].

The first recognized instance of prion disease crossing the species barrier to humans occurred in the United Kingdom in the 1990s, where variant bovine spongiform encephalopathy caused an epidemic from contamination of meat and bone meal containing scrapie, which was then accelerated by recycling infected bovine tissues [12]. Humans who consumed beef products from prion-infected cattle during this period were diagnosed with CJD. Recently, in wild and captive cervids, CWD has been increasing in frequency, raising concerns about managing this disease in wildlife species such as deer and elk [5,13]. Although transmission between cervids and humans has not yet been documented, there is a possible risk to human health in handling and consuming the potentially asymptomatic deer tissue [14,15].

Some mammalian species have been reported to possess varying levels of resistance to prion disease. For example, members of the mammalian families Canidae [16,17,18,19,20,21,22,23,24,25], Equidae [24,26,27,28,29], Leporidae [30,31], Mustelidae [20], and Suidae [32] have been shown to be highly resistant to prion diseases [33,34,35,36]. New Zealand White rabbits (*Oryctolagus cuniculus*) displayed a low susceptibility to prion disease demonstrated by in vitro experiments [37,38], whereas the prion protein associated with horses (*Equus caballus*) and dogs (*Canis familiaris*), respectively, were unaffected by these inoculation trials, indicating horses and dogs do not naturally contract the disease and subsequently are more resistant to the change in protein conformation [24,28,35].

Recent studies demonstrated that most members of Suborder Ruminantia (cattle, deer, goats, sheep, and allies) are highly susceptible to prion diseases, with codon positions at 136, 154, and 171 serving as important codons leading to the susceptibility of sheep to scrapie [39,40,41,42]. Some domestic sheep species with the R1 classification (genotype A136R154R171/A136R154R171) have been deliberately selected to be resistant to scrapie [6,43]. In addition, there is evidence suggesting that water buffalo (*Bubalus bubalis*) have low-level susceptibility to TSEs similar to that reported for rabbits, dogs, and horses [44].

Although the hypothesis of truly resistant mammals to prion diseases is contentious [37,45,46,47,48,49,50,51], mammals may have a low susceptibility to prion diseases based on data accrued from in vitro and in vivo challenges under highly favorable laboratory conditions, but never encounter or develop prion disease in nature [24,34,35]. Polymenidou et al. [17] explored the risk of replicating prions in Madin Darby canine kidney (MDCK, originally derived from *Canis familiaris*, breed: cocker spaniel) 33,016 cells, wherein these cells failed to induce prion infection using different strains of prion diseases. Polymenidou et al. [17] indicated that although there was no direct evidence that the canine PrP^C^ (cPrP^C^) can convert into a pathological isoform, it is reasonable to assume they have been exposed to BSE-contaminated feed, and therefore probably are immune to infection. Further, Polymenidou et al. [20] reported low levels of expressed cPrP^C^ and that it was not located on raft fractions, which was hypothesized to contribute to MDCK cells possessing an apparent resistance to prions. Further, Lysek et al. [16] used nuclear magnetic resonance (NMR) analyses to determine that the presence of D159 (numbering based on human *PRNP*) and R177 (numbering based on human *PRNP*) in dogs (*C. familiaris*) causes unique charge distribution patterns on the front and back side of cPrP^C^ when compared to sheep and feline PrP. This variation in cPrP^C^ structure may also correlate with the protection of *C. familiaris* against challenge with BSE. In addition, cPrP^C^ possesses stable molecular structures under neutral and low pH environments [19].

In an attempt to discover the molecular basis for resistance, Stewart et al. [20] examined the PrP^C^ sequences of 18 species, representative of the Suborder Caniformia, in which sequences representative of members of Canidae and Mustelidae possessed two substitutions (aspartic acid—D or glutamic acid—E) at codon site D/E163 (numbering based on *C. familiaris PRNP*). PrP^C^ sequences of felids did not contain this codon (possessed asparagine—N instead); therefore, Stewart et al. [20] proposed that D/E163 provided a genetic basis for observed susceptibility differences between members of Caniformia and Feliformia. Fernandez-Borges et al. [22] further confirmed the significance of D163 using in vitro and in vivo analyses, involving a transgenic mouse model that demonstrated complete resistance to mouse prion strains despite intracerebral exposure. Further, D163 from cPrP^C^ was demonstrated to have no effect in *Drosophila*; whereas N163 cPrP^C^ caused robust neurodegenerative changes in *Drosophila*, indicating that D163 is a protective residue that provides intrinsic resistance to toxic conformations and increases the overall conformational stability of cPrP^C^ [21,24].

Although the residue D163 initially was thought to be exclusive to canids, anecdotal evidence suggests that this nonsynonymous substitution appears in some chiropteran (bats) species [9,20,22]; possibly as a result of genetic drift [20]. To date, no natural or experimental prion diseases have been documented in bat species. For example, a recent study by Eraña et al. [42] provided a substantial number of prion protein gene sequences and examined the processes involved in the reactivity/conversion to misfolded proteins; however, they did not examine this in any of the dogs or bats with the D/E163 substitutions. Therefore, the goals of this study were to: (1) obtain DNA sequences for the *PRNP* gene from additional representatives of Chiroptera to comprehensively determine if the presence of substitutions at codon position 163 conferred a potential for susceptibility as was reported in canids, (2) survey (i.e., data mine) and compare all reported *PRNP* sequences (from NCBI GenBank) to those responsible for generating diseases to identify potential patterns of susceptibility or resistance, and (3), determine if DNA sequences from the *PRNP* gene in mammalian species provided evidence for patterns of molecular evolution that reflected either a gene tree or species tree.

## 2. Materials and Methods

### 2.1. Sampling

A total of 20 tissue samples (see Supplemental Data Table S1 for species associated with NCBI GenBank accession numbers PQ577305–PQ577324), representing the suborder Caniformia (*n* = 8) and order Chiroptera (*n* = 12), were obtained through a destructive loan process from the Robert J. Baker Genetic Resource Collection housed in the Natural Science Research Laboratory (NSRL), Museum at Texas Tech University. These samples were obtained from wild-caught individuals at various localities in Texas and Louisiana. To expand the taxonomic coverage, all prion protein gene nucleotide sequences were retrieved from NCBI GenBank (*n* = 12,337), and the following were filtered and removed: all genome shotgun sequences and genes for the shadow of prion protein (*SPRN*), prion-like doppel (*PRND*), among other genes. The filtered dataset was then reduced using RAxML [52], which identifies identical sequences and removes them, and a visual assessment was performed, resulting in a final dataset of 862 sequences (see Supplemental Data Table S1). Visual assessment involved screening all sequences for the codon of interest. If the codon of interest was not observed in a species, then one complete sequence was chosen at random to represent the species.

### 2.2. DNA Sequencing

Genomic DNA (gDNA) for 20 samples representing species in Caniformia and Chiroptera were isolated from 0.1g of liver or muscle tissue using the Qiagen DNeasy kit (Qiagen, Valencia, CA, USA). For all samples, the full-length *PRNP* gene (742–811 bp) was amplified using PCR (polymerase chain reaction, [53]) with the amplification primers: PRNP_F (forward, 5′-ATGGTGAAAAGCCACATAGGCGGC-3′) and PRNP_R (reverse, 5′-TCATCCCACTATCARGARAATGAG-3′). PCR reactions contained 12.5 µL HotStarTaq (Qiagen, Valencia, CA, USA), 8.3 µL distilled H_2_O, 0.6 µL of each primer, and 3 µL gDNA. The thermal profile parameters are as follows: hot start of 80 °C, initial denaturation at 95 °C for 2 min, followed by 34 cycles of denaturation at 95 °C for 30 s, annealing at a range of 52–54 °C for 45 s, extension at 73 °C for 1 min, and a final extension at 73 °C for 15 min.

All PCR products were purified with ExoSAP-IT (Applied Biosystems, Foster City, CA, USA). Cycle sequencing reactions used 5 µL of purified PCR products from the previous step, 2 µL ABI Prism Big Dye version 3 terminator ready reaction and buffer master mix (Applied Biosystems, Foster City, CA, USA), and 3 µL 1 µM of PRNP_F and PRNP_R, respectively. Sequencing reactions were purified using Sephadex columns (Cytiva, Marlborough, MA, USA) and centrifugation, followed by dehydration. Purified products were analyzed on an ABI 3730xl automated sequencer (Eurofins Genomics LLC, Louisville, KY, USA). Resulting sequences were aligned and proofed with Sequencher 4.10.1 software (Gene Codes Corporation, Ann Arbor, MI, USA), and chromatograms were inspected to authenticate any base changes.

### 2.3. Phylogenetic Analyses

A total of 882 *PRNP* sequences (20 generated herein and 862 obtained from NCBI GenBank) were used to construct a mammalian phylogeny. The platypus (*Ornithorhynchus anatinus*, EU559338) and echidna (*Tachyglossus aculeatus*, BK063950) were designated as outgroup taxa. Duplicate species were removed unless the sequence was representative of a species belonging to Caniformia or Chiroptera. For example, >900 sequences were returned when “*Ovis aries* prion protein gene” was queried on NCBI GenBank and a randomly chosen complete sequence was used as a representative for this species. Further, when querying for prion protein genes, other genes (shadow of prion protein, *SPRN*; prion-like doppel gene, *PRND*) were invariably included in the search and eliminated before processing the data. Once all non-target genes were removed, all remaining *PRNP* sequences were parsed into respective orders, trimmed to only include the *PRNP* gene, and aligned by their protein sequence to maintain the structure of the prion protein. Then all orders were assimilated and aligned by their protein sequence (if the nucleotide sequences were aligned, then the amino acid structure would be altered), resulting in an alignment of 1245 bp, which excluded stop codons. This final dataset was used for all downstream analyses.

Eighty-eight maximum likelihood (ML) models were evaluated using jModelTest-−2.1.10 [54,55]. The Akaike information criterion with a correction for finite sample sizes (AICc, [56,57]) identified the TPM2uf+I+G model of evolution (-lnL = 49,137.9405) as the most appropriate for the *PRNP* dataset. However, the general time reversible [58] plus proportion of invariable sites plus gamma distribution (GTR+I+Γ) model of nucleotide substitution, the most complex model, has been suggested to fit complex datasets better than simpler models [59,60,61]; therefore, we proceeded with the GTR+I+Γ model for all analyses.

A Bayesian inference (BI) model (MrBayes v3.2.6, [62]) was conducted to generate a phylogenetic tree under an ML framework and to generate posterior probability values (PPV) indicating nodal support. The GTR+I+Γ nucleotide substitution model and the following parameters were used: two independent runs with four Markov chains (one cold and three heated; MCMCMC), 10 million generations, and a sample frequency of every 1000 generations from the last nine million generated. A visual inspection of likelihood scores resulted in the first 1,000,000 trees being discarded (10% burn-in) and a consensus tree (50% majority rule) constructed from the remaining trees. PPV ≥ 0.95 was used to designate nodal support [63].

### 2.4. Evaluation of a Targeted Amino Acid Substitution

The program MEGA11 [64] was used to translate the nucleotide sequences to amino acids, allowing for the detection of any non-synonymous substitutions in this region. A series of 16 amino acids (DYEDRYYRENMHRYPN) based on the site of interest (N159D/E) was targeted to document the occurrence of aspartic or glutamic acids across mammalian taxa.

## 3. Results

### 3.1. Phylogenetic Analyses

**Figure 1:**
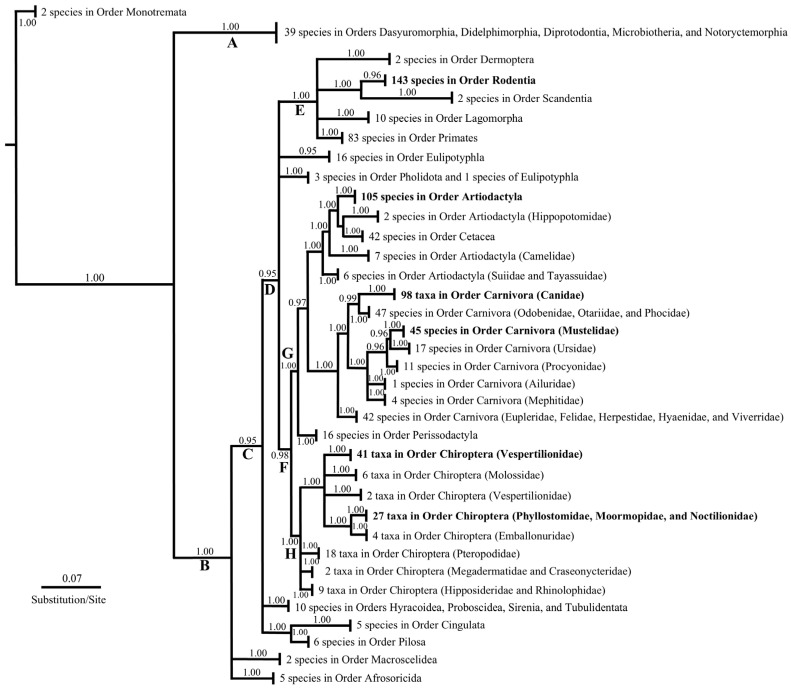
Phylogeny of the exon 3 region of the prion protein gene using 882 mammalian sequences. Bayesian posterior probability values represent ≥95% nodal support. Phylogenetic groups shown in bold text indicate the occurrence of N159D/E.

A total of 882 sequences (1245 bp in length) representing 26 Orders, 132 families, and 686 species were used to recover patterns of molecular evolution in the *PRNP* gene. The Bayesian Inference analysis (Figure 1) produced a topology that was largely unresolved at the ordinal and familial levels, as most basal nodes were collapsed due to a lack of statistical support. Eight major Clades A–H (A, 5 Metatherian orders), (B, 20 Eutherian orders), (C, 19 Eutherian orders excluding Macroscelidea and Afrosoricidea), (D, Dermoptera, Rodentia, Scandentia, Lagomorpha, Primates, Eulipotyphla, Cetartiodactyla (Cetacea and Artiodactyla), Carnivora, Perissodactyla, and Chiroptera), (E, Dermoptera, Rodentia, Scandentia, Lagomorpha, and Primates), (F, Cetartiodactyla, Carnivora, Perissodactyla, and Chiroptera), (G, Cetartiodactyla and Carnivora), and (H, Chiroptera) were identified as possessing supported relationships. Phylogenetic placement of the orders Eulipotyphla, Tubulidentata, Hyracoidea, Sirenia, Proboscidea, Pilosa, Cingulata, Macroscelidea, and Afrosoricidea was largely unresolved, as noted by their ethereal inclusion in Clades B, C, and D.

### 3.2. Evaluation of a Targeted Amino Acid Substitution

Across the Mammalian orders included herein, only Carnivora, Chiroptera, Artiodactyla, and Rodentia contained species that possessed aspartic acid and/or glutamic acid at codon position 302 (D302 or E302, respectively, Table 1 and Table 2). It should be noted that codon position 159 in humans and 163 in dogs corresponds to codon position 302 as discussed herein as a result of aligning the additional mammalian sequences, which possessed varying numbers of octapeptide repeats. With the exception of the primate *Loris tardigradus* (glycine at 302, G302), all other mammal species possessed either an asparagine or serine at 302 (N302 or S302, respectively). Expanding on Stewart et al. [20], within Caniformia, 41 species in Canidae and Mustelidae were monomorphic for aspartic acid at site 302 (D302); with the only exception being several breeds of domestic dogs (*Canis familiaris*, polymorphic at codon 302—D/E302). Other Caniformia families (Ursidae, Odobenidae, Otariidae, Phocidae, Ailuridae, Procyonidae, Mephitidae) and Feliformia families (Eupleridae, Felidae, Herpestidae, Hyaenidae, and Viverridae) were monomorphic for N302. Mephitidae, which is sister to the Mustelidae [65], did not possess the D302 substitution. Further, 23 species of bats represented by Mormoopidae and Vespertilionidae also possessed D/E302. Unexpectedly, one species of Moschidae and two species of Erethizontidae were characterized by D302.

Within Chiroptera, only Mormoopidae (2 species) and Vespertilionidae (2 species) were shown to possess species containing the D302 substitution; the other 19 species of Chiroptera possessed the E302 codon (Table 1 and Table 2). In Artiodactyla, of the 9 families (117 species) examined, only one of two species in the family Moschidae (*Moschus moschiferus*) possessed the D302 codon. In Rodentia, only *Coendou prehensilis* and *Erethizon dorsatum* possessed codon D302, with the remaining 141 species possessing the N302 or S302 substitutions. All other mammalian groups, including rabbits, horses, and pigs, which have low susceptibility to prion diseases [44], did not possess the D or E codon.

## 4. Discussion

Our broad-based examination of 882 *PRNP* sequences revealed that only 53 of the 686 species (including 1 domesticated species, *Canis familiaris*, and 52 wild species of mammals) possessed either the aspartic or glutamic codon substitution (Figure 1, Table 1 and Table 2). Of these, 27 species were associated with the Carnivora (Canidae and Mustelidae); 23 species with Chiroptera (Mormoopidae and Vespertilionidae), one species of Artiodactyla (Moschidae–*Moschus moschiferus*), and two species of Rodentia (Erethizontidae–*Coendou prehensilis* and *Erethizon dorsatum*). Therefore, our dataset demonstrates that *C. familiaris* is not the only mammalian species with codon D/E159 and provides support for select, wild members of Artiodactyla, Caniformia, Chiroptera, and Rodentia to potentially possess an innate resistance to prion diseases.

The dataset examined herein (686 species) lacks sufficient taxonomic coverage (proposed 6495 mammalian species, [66]) to definitively establish any meaningful association between the aspartic acid or glutamic acid residue at human site 159, and resistance to prion diseases. However, where taxonomic coverage was sound, the large percentage of individuals lacking either of the resistant substitutions argues against inadequate sampling as a reason. Consequently, we offer four independent scenarios to potentially address these unexpected results.

**Phylogenetic patterns revealed that the distribution of D/E159 was clearly not the product of shared ancestral/descendant relationships and provides poor evidence for a single origin involving the appearance of resistance; therefore, it may be the product of an unknown mechanism driving convergent evolution.**

Phylogenetic analyses (Figure 1) depicted weak support among major groups, resulting in the “collapsing” of several branches/clades at basal nodes. For example, only certain members of the Carnivora, Chiroptera, Artiodactyla, and Rodentia possessed the amino acid substitution of interest, providing the appearance of a “random” distribution of resistance at the ordinal level. Further, the fact that these four orders are not closely related and lack phylogenetic signal, from a phylogenetic context and comparing Figure 1 to established mammalian speciation phylogenies (see [67,68,69,70] for a summary of mammalian phylogenetic relationships), supports the possibility of codon convergence in response to prion disease activity and indicates a propensity for the *PRNP* sequences to reflect a specific gene tree pattern of molecular evolution rather than reflecting phylogenetic relationships. In other words, it appears that the proposed resistance at human site 159 (canid site 163, site 302 herein) identified in Stewart et al. [20] is not distributed across mammalian orders due to a shared ancestry. Indeed, its presence in only a few individuals scattered across the distantly related groups, Carnivora, Chiroptera, Artiodactyla, and Rodentia, indicates that a shared evolutionary history from a common ancestor is unlikely and that an independent or repeated origin is most likely.

**If D/E159 were the evolutionary products of a dietary response, then protection from consuming prion-infected prey or carrion would be expected in the PrP sequence of obligative carnivores (i.e., felids)**

The occurrence of resistant codons across phylogenetically unrelated taxa may be the result of a molecular response to diets involving the consumption of tissue infected with prion disease. Of the 14 of 15 recognized Carnivora families (Supplemental Data Table S1; data were not available for Nandinidae) examined herein, only Canidae and Mustelidae possessed D/E159. By extension, the presence of the D/E159 substitution in wild members of the Canidae and Mustelidae could be an evolutionary adaptive form of protection against exposure to prion diseases such as CWD. For example, coyotes remain uninfected after consuming CWD-positive brain homogenates from elk, although prions from the homogenate are present in the coyote’s feces [71]. Further, Onizuka [18] suggested that herbivores possessed less resistance to prion diseases, probably because they are less exposed to prion transmission as opposed to carnivores.

The above diet hypothesis may explain the appearance of D/E159 for Canidae and Mustelidae; however, it does not explain the absence of D/E159 in other families of Caniformia (diet includes fresh flesh and scavenging) or Feliformia, which almost exclusively consume fresh flesh. One could assume that some members of Carnivora would never encounter CWD-infected individuals, considering some are omnivores (e.g., Procyonidae and Ailuridae) and others mainly inhabit aquatic environments (e.g., Odobenidae, Otariidae, and Phocidae). Members of Feliformia are obligate carnivores to some extent, yet these mammals do not possess D/E159. Hyaenidae presents an exceptional case, considering similar adaptations to canids (e.g., morphology, social hierarchy, hunting strategies, etc.) and specialization in carrion consumption.

Further, the occurrence of “resistant codons” scattered across the phylogenetically unrelated Chiroptera, Artiodactyla, and Rodentia requires alternative hypotheses. For example, the presence of D/E159 in Chiroptera may have evolved as a need for prion resistance due to their insectivorous diet. During the ingestion of insects, it is possible that bats consume adult insects that were infected during larval stages. However, even this explanation is complicated, as only two of 10 species in Mormoopidae and only 19 of 400+ species in the Vespertilionidae possessed the codon. Although most species in these two phylogenetically distant families are insectivores, the occurrence of the D/E159 in only 23 species does not suggest a strong correlation between the presence of this codon and an insectivorous diet. In addition, the fact that insectivory arose multiple times independently across Chiroptera infers a pattern of convergent evolution. Further, the geographic regions of Vespertilionid species with the substitution predominantly include not only North America, but also portions of Eurasia. The geographic region of non-substituted individuals also includes North America and Eurasia, with the observed overlap indicating no geographical significance. Although resistance to prion disease in Chiroptera has been hypothesized based on the presence of D/E159 [9,20], genotypic and in vivo challenges need to be explored in further studies to determine the breadth of resistance among taxa representative of Chiroptera and the corresponding potential resistance to prion diseases. Additionally, the presence of D159 in species (*Moschus moschiferus*, *Coendou prehensilis*, and *Erethizon dorsatum*) restricted to herbivory does not fit any dietary model that could explain the evolutionary need for protection against prion diseases.

Overall, the presence of N159 in 633 of the examined 686 mammalian species, especially those with carnivorous diets, invalidates the hypothesis of diet as a selective pressure and leans in favor of convergent evolution as the selective pressure that drove acquisition of N159D/E in Canidae and Mustelidae (Carnivora), Mormoopidae and Vespertilionidae (Chiroptera), Moschidae (Artiodactyla), and Erethrizontidae (Rodentia).

**Effects of additional codon substitutions among mammals**

N159D/E is not necessarily the only substitution that could confer resistance. It may be that additional codon sites play an additive role with D/E159, or that the repeat areas in the *PRNP* gene increase the opportunities for mutational hotspots. For example, several studies have examined breeds of dogs or species of wild canids and have found that polymorphisms at position canid N107S and R/C181H of cPrP^C^ for Pekingese [72], *Vulpes lagopus* [73], *Vulpes corsac* [74], and *Nyctereutes procyonoides* [75] are consistent with polymorphisms seen in other canid species but are not shared by other mammalian species. Further, at codon 303 based on the alignment generated herein, all mammals have a Q, whereas *Antrozous pallidus* (BK064069), *Lasionycteris noctivaga* (TK94660), and *Ia io* (BK064208) possessed Q303H and *Eptesicus fuscus* (BK064945, XM054723678) and *Eptesicus nilssonii* (BK064950) possessed Q303R.

**Possibility of complete resistance in the natural environment**

It is unclear whether mammalian species can be broadly resistant to different TSEs. In other studies, D/E159 associated with canids did not provide complete protection against a BSE prion infection [11]. Further, when transgenic mice were inoculated with BSE and a classical scrapie strain, they displayed resistance [11,25], but exposure to bovine atypical L-BSE and other non-classical scrapie isolates resulted in susceptibility. In addition, some BSE isolates displayed a shift in their prion strain properties; therefore, it may be that a combination of different amino acid residues or possibly genes other than *PRNP* is needed to provide a protective effect against ruminant prions [11].

When considering the structure of cPrP, it is apparent that D159 is located on the α1-β2 loop and thereby provides structural stability and prolongs the period in prion-infected transgenic mice carrying this allele [22,23]. Further, transgenic mouse models have shown resistance to prion strains, including SSBP/1, BSE-C, CWD, sheep-BSE, cat CWD, BSE-L and atypical scrapie and demonstrated that D/E159 is protective because the alternative codon substitution susceptibility to prion transmission is increased in mice expressing cPrP-D159N [25]. Although the reverse substitution (cPrP-N159D) is not protective in human PrP [76], this substitution (cPrP-N159D) and cPrP-H177R inhibited liquid–liquid phase separation and amyloid production in human PrP^C^ [77]. In addition, linkage disequilibrium (LD) between *PRNP* and prion-like protein (Doppel) gene (*PRND*) may play a factor in susceptibility. For example, Mesquita et al. [78] and Jeong et al. [79] demonstrated strong LD between *PRNP* and *PRND* in sheep and goats, respectively, whereas Won et al. [80,81] found evidence of a weak LD between *PRNP* and *PRND* in dogs and horses.

## 5. Conclusions

It is important to note that these hypotheses do not explain the level of resistance observed in all of the 53 examined species possessing the D/E159 substitution. It could be a combination of these, or even an unknown hypothesis is at play. However, it is evident based on available sequence data that the D/E159 substitution is not a product of domestication and exists in wild mammalian species. Therefore, to investigate the breadth of resistance of D/E159 of PrP, researchers need to expand inoculation trials and knock-in experiments in mice [22,37,82], as well as other studies to address sequence compatibility, mechanism of onset, and comparative models, to include the 53 species of the mammalian groups identified herein (canids, mormoopids and vesper bats, musk deer, and some rodents). These types of experiments would be highly informative to further understand the true nature of codon D/E159 beyond its documented in vitro resistance in dog breeds. For example, if future knock-in studies demonstrate a continued resistance pattern to prion diseases in vitro for these other 53 mammalian species examined herein, then the impact of those studies would provide a substantial contribution toward developing mechanistic solutions to modifying prion sensitivity in managed mammalian species. For example, the future of deer breeding operations may rely on CRISPR and cloning techniques using D/E159 as a guide to effectively prevent CWD in managed, captive deer populations and thereby greatly reduce CWD outbreaks in nearby wild deer populations and the economic loss of culling entire herds within a deer breeding operation.

## Figures and Tables

**Table 1:** All mammal species, except those listed below, possess an asparagine (N) at codon 159. Within the Order Carnivora and Chiroptera, there were a few cases where certain species possess the codon substitution from asparagine to aspartic (D) or glutamic (E) acids. Sample size refers to the number of available sequences, not the number of species.

Order	Family	Species	Sample Size	Amino Acid
Carnivora	Canidae	Domestic dog and dingo, *Canis familiaris*	66	D/E
		Coyote, *C. latrans*	5	D
		Grey wolf, *C. lupus*	4	D
		Maned wolf, *Chrysocyon brachyurus*	1	D
		African wild dog, *Lycaon pictus*	1	D
		Side-striped jackal, *Lupulella adustus*	1	D
		Black-backed jackal, *L. mesomelas*	1	D
		Common raccoon dog, *Nyctereutes procyonoides*	4	D
		Bat-eared fox, *Otocyon megalotis*	1	D
		Bush dog, *Speothos venaticus*	1	D
		Grey Fox, *Urocyon cinereoargenteus*	2	D
		Island fox, *U. littoralis*	1	D
		Corsac fox, *V. corsac*	1	D
		Tibetan fox, *Vulpes ferrilata*	1	D
		Arctic fox, *V. lagopus*	3	D
		Swift fox, *V. velox*	1	D
		Red fox, *Vulpes vulpes*	4	D
		Fennec fox, *V. zerda*	1	D
	Mustelidae	Tayra, *Eira barbara*	1	D
		Wolverine, *Gulo gulo*	3	D
		Yellow-throated marten, *Martes flavigula*	1	D
		Beech marten, *M. foina*	1	D
		European pine marten, *M. martes*	1	D
		Sable, *M. zibellina*	1	D
		Honey badger, *Mellivora capensis*	1	D
		Fisher, *Pekania pennanti*	1	D
		American badger, *Taxidea taxus*	2	D
Chiroptera	Mormoopidae	Mesoamerican common mustached bat, *Pteronotus mesoamericanus*	2	D
		Parnell’s mustached bat, *P. parnellii*	1	D
	Vespertilionidae	Hoary bat, *Aeorestes cinereus*	2	E
		Pallid bat, *Antrozous pallidus*	1	E
		Rafinesque’s big-eared bat, *Corynorhinus rafinesquii*	2	E
		Townsend’s big-eared bat, *C. townsendii*	2	E
		Western yellow bat, *Dasypterus xanthinus*	2	E
		Big brown bat, *Eptesicus fuscus*	2	E
		Northern bat, *E. nilssonii*	1	E
		Great evening bat, *Ia io*	1	E
		Silver-haired bat, *Lasionycteris noctivaga*	1	E
		Eastern red bat, *Lasiurus borealis*	2	E
		Western red bat, *L. frantzi*	1	E
		Brandt’s bat, *Myotis brandtii*	1	D
		Little brown bat, *M. lucifugus*	2	D
		Birdlike noctule, *Nyctalus aviator*	1	E
		Evening bat, *Nycticeius humeralis*	1	E
		Canyon bat, *Parastrellus hesperus*	1	E
		Tricolored bat, *Perimyotis flavus*	1	E
		Kuhl’s pipistrelle, *Pipistrellus kuhlii*	2	E
		Common pipistrelle, *P. pipistrellus*	2	E
		Brown long-eared bat, *Plecotus auritus*	1	E
		Parti-colored bat, *Vespertilio murinus*	1	E
Artiodactyla	Moschidae	Siberian musk deer, *Moschus moschiferus*	1	D
Rodentia	Erethizontidae	Brazilian porcupine, *Coendou prehensilis*	1	D
		North American porcupine, *Erethizon dorsatum*	1	D

**Table 2:** Sequence alignments of the 53 species characterized by D/E159 (in bold) using the human prion protein (PrP) sequence as a reference. Dashes represent no change from the human PrP sequence.

	Species	144	145	146	147	148	149	150	151	152	153	154	155	156	157	158	159	160	161
1	Human, *Homo sapiens*	D	Y	E	D	R	Y	Y	R	E	N	M	H	R	Y	P	**N**	Q	V
2	Domestic dog and dingo, *Canis familiaris*	-	-	-	-	-	-	-	-	-	-	-	Y	-	-	-	**D**	-	-
3	Coyote, *C. latrans*	-	-	-	-	-	-	-	-	-	-	-	Y	-	-	-	**D**	-	-
4	Grey wolf, *C. lupus*	-	-	-	-	-	-	-	-	-	-	-	Y	-	-	-	**D**	-	-
5	Maned wolf, *Chrysocyon brachyurus*	-	-	-	-	-	-	-	-	-	-	-	Y	-	-	-	**D**	-	-
6	African wild dog, *Lycaon pictus*	-	-	-	-	-	-	-	-	-	-	-	Y	-	-	-	**D**	-	-
7	Side-striped jackal, *Lupulella adustus*	-	-	-	-	-	-	-	-	-	-	-	Y	-	-	-	**D**	-	-
8	Black-backed jackal, *L. mesomelas*	-	-	-	-	-	-	-	-	-	-	-	Y	-	-	-	**D**	-	-
9	Common raccoon dog, *Nyctereutes procyonoides*	-	-	-	-	-	-	-	-	-	-	-	Y	-	-	-	**D**	-	-
10	Bat-eared fox, *Otocyon megalotis*	-	-	-	-	-	-	-	-	-	-	-	Y	-	-	-	**D**	-	-
11	Bush dog, *Speothos venaticus*	-	-	-	-	-	-	-	-	-	-	-	Y	-	-	-	**D**	-	-
12	Grey Fox, *Urocyon cinereoargenteus*	-	-	-	-	-	-	-	-	-	-	-	Y	-	-	-	**D**	-	-
13	Island fox, *U. littoralis*	-	-	-	-	-	-	-	-	-	-	-	Y	-	-	-	**D**	-	-
14	Corsac fox, *Vulpes corsac*	-	-	-	-	-	-	-	-	-	-	-	Y	-	-	-	**D**	-	-
15	Tibetan fox, *V. ferrilata*	-	-	-	-	-	-	-	-	-	-	-	Y	-	-	-	**D**	-	-
16	Arctic fox, *V. lagopus*	-	-	-	-	-	-	-	-	-	-	-	Y	-	-	-	**D**	-	-
17	Swift fox, *V. velox*	-	-	-	-	-	-	-	-	-	-	-	Y	-	-	-	**D**	-	-
18	Red fox, *V. vulpes*	-	-	-	-	-	-	-	-	-	-	-	Y	-	-	-	**D**	-	-
19	Fennec fox, *V. zerda*	-	-	-	-	-	-	-	-	-	-	-	Y	-	-	-	**D**	-	-
20	Tayra, *Eira barbara*	-	-	-	-	-	-	-	-	-	-	-	-	-	-	-	**D**	-	-
21	Wolverine, *Gulo gulo*	-	-	-	-	-	-	-	-	-	-	-	-	-	-	-	**D**	-	-
22	Yellow-throated marten, *Martes flavigula*	-	-	-	-	-	-	-	-	-	-	-	-	-	-	-	**D**	-	-
23	Beech marten, *M. foina*	-	-	-	-	-	-	-	-	-	-	-	-	-	-	-	**D**	-	-
24	European pine marten, *M. martes*	-	-	-	-	-	-	-	-	-	-	-	-	-	-	-	**D**	-	-
25	Sable, *M. zibellina*	-	-	-	-	-	-	-	-	-	-	-	-	-	-	-	**D**	-	-
26	Honey badger, *Mellivora capensis*	-	-	-	-	-	-	-	-	-	-	-	-	-	-	-	**D**	-	-
27	Fisher, *Pekania pennanti*	-	-	-	-	-	-	-	-	-	-	-	-	-	-	-	**D**	-	-
28	American badger, *Taxidea taxus*	-	-	-	-	-	-	-	-	-	-	-	-	-	-	-	**D**	-	-
29	Mesoamerican common mustached bat, *Pteronotus mesoamericanus*	-	-	-	-	-	-	-	-	-	-	-	-	-	F	-	**D**	-	-
30	Parnell’s mustached bat, *P. parnellii*	-	-	-	-	-	-	-	-	-	-	-	-	-	F	-	**D**	-	-
31	Hoary bat, Aeorestes cinereus	E	-	-	-	-	-	-	-	-	-	-	N	-	F	-	**E**	-	-
32	Pallid bat, *Antrozous pallidus*	E	-	-	-	-	-	-	-	-	-	-	N	-	F	-	**E**	H	-
33	Rafinesque’s big-eared bat, *Corynorhinus rafinesquii*	E	-	-	-	-	-	-	-	-	-	-	N	-	F	-	**E**	-	-
34	Townsend’s big-eared bat, *C. townsendii*	E	-	-	-	-	-	-	-	-	-	-	N	-	F	-	**E**	-	-
35	Western yellow bat, *Dasypterus xanthinus*	E	-	-	-	-	-	-	-	-	-	-	N	-	F	-	**E**	-	-
36	Big brown bat, *Eptesicus fuscus*	E	-	-	-	-	-	-	-	-	-	-	N	-	F	-	**E**	R	-
37	Northern bat, *E. nilssonii*	E	-	-	-	-	-	-	-	-	-	-	N	-	F	-	**E**	R	-
38	Great evening bat, *Ia io*	E	-	-	-	-	-	-	-	-	-	-	N	-	F	-	**E**	H	-
39	Silver-haired bat, *Lasionycteris noctivaga*	E	-	-	-	-	-	-	-	-	-	-	N	-	F	-	**E**	H	-
40	Eastern red bat, *Lasiurus borealis*	E	-	-	-	-	-	-	-	-	-	-	N	-	F	-	**E**	-	-
41	Western red bat, *L. frantzi*	E	-	-	-	-	-	-	-	-	-	-	N	-	F	-	**E**	-	-
42	Brandt’s bat, *Myotis brandtii*	E	-	-	-	-	-	-	-	-	-	-	N	-	F	-	**D**	-	-
43	Little brown bat, *M. lucifugus*	E	-	-	-	-	-	-	-	-	-	-	N	-	F	-	**D**	-	-
44	Birdlike noctule, *Nyctalus aviator*	E	-	-	-	-	-	-	-	-	-	-	N	-	F	-	**E**	-	-
45	Evening bat, *Nycticeius humeralis*	E	-	-	-	-	-	-	-	-	-	-	N	-	F	-	**E**	-	-
46	Canyon bat, *Parastrellus hesperus*	E	-	-	-	-	-	-	-	-	-	-	N	-	F	-	**E**	-	-
47	Tricolored bat, *Perimyotis flavus*	E	-	-	-	-	-	-	-	-	-	-	N	-	F	-	**E**	-	-
48	Kuhl’s pipistrelle, *Pipistrellus kuhlii*	E	-	-	-	-	-	-	-	-	-	-	N	-	F	-	**E**	-	-
49	Common pipistrelle, *P. pipistrellus*	E	-	-	-	-	-	-	-	-	-	-	N	-	F	-	**E**	-	-
50	Brown long-eared bat, *Plecotus auritus*	E	-	-	-	-	-	-	-	-	-	-	N	-	F	-	**E**	-	-
51	Parti-colored bat, *Vespertilio murinus*	E	-	-	-	-	-	-	-	-	-	-	N	-	F	-	**E**	-	-
52	Siberian musk deer, *Moschus moschiferus*	-	-	-	-	-	-	-	-	-	-	-	Y	-	-	-	**D**	-	-
53	Brazilian porcupine, *Coendou prehensilis*	-	-	-	-	-	-	-	-	-	-	-	Y	-	-	-	**D**	-	-
54	North American porcupine, *Erethizon dorsatum*	-	-	-	-	-	-	-	-	-	-	-	Y	-	-	-	**D**	-	-

## Data Availability

All sequence data generated for this paper were accessioned at NCBI GenBank under PQ577305–PQ577324.

## Supplementary Materials

The following supporting information can be downloaded at: https://www.mdpi.com/article/10.3390/ani16071059/s1, Table S1: All examined sequences with their associated taxonomic classifications and NCBI GenBank accession numbers.

## Abbreviations

The following abbreviations are used in this manuscript:

TSE	Transmissible spongiform encephalopathies
PRNP	Exon 3 region of the prion protein gene
SE	Spongiform encephalopathies
CJD	Creutzfeldt-Jakob disease
GSS	Gertsmann-Sträussler-Scheinker disease
VPSPr	Variable protease-sensitive prionopathy
CWD	Chronic wasting disease
BSE	Bovine spongiform encephalopathy
TME	Transmissible mink encephalopathy
FSE	Feline spongiform encephalopathy
CPD	Camel prion disease
PrP	Prion protein
PrP^C^	Normal cellular PrP
PrP^Sc^	Infectious conformation of PrP
MDCK	Madin Darby canine kidney
cPrP^C^	canine PrP^C^
NMR	Nuclear magnetic resonance
DNA	Deoxyribonucleic acid
gDNA	Genomic DNA
PCR	Polymerase chain reaction
ML	Maximum likelihood
AICc	Akaike information criterion with a correction
GTR+I+Γ	general time reversible plus proportion of invariable sites plus gamma distribution
BI	Bayesian inference
PPV	Posterior probability values
